# MiR-378 mediates the ovariectomy induced bone loss via exaggerating osteoclastogenesis and transforming growth factor beta impaired osteogenesis

**DOI:** 10.1016/j.gendis.2025.101754

**Published:** 2025-07-03

**Authors:** Lu Feng, Zhengmeng Yang, Nan Hou, Haixing Wang, Shanshan Bai, Xuan Lu, Yaofeng Wang, Sien Lin, Micky D. Tortorella, Gang Li

**Affiliations:** a Centre for Regenerative Medicine and Health, Hong Kong Institute of Science & Innovation, Chinese Academy of Sciences, Hong Kong SAR, China; b Stem Cells and Regenerative Medicine Laboratory, Li Ka Shing Institute of Health Sciences, The Chinese University of Hong Kong, Prince of Wales Hospital, Shatin, Hong Kong SAR, China; c Musculoskeletal Research Laboratory, Department of Orthopaedics & Traumatology, Faculty of Medicine, The Chinese University of Hong Kong, Prince of Wales Hospital, Shatin, Hong Kong SAR, China; d Institute of Biomedicine and Biotechnology, Shenzhen Institute of Advanced Technology, Chinese Academy of Sciences, Shenzhen, Guangdong 518055, China

**Keywords:** Inflammation, miR-378, Osteoclastogenesis, Osteogenesis, Osteoporosis

## Abstract

Osteoporosis (OP) is a disease characterized by decreased bone mass and damaged architectures. The promising treatment strategy for OP is to inhibit bone resorption while promoting bone formation. MicroRNAs (miRNAs) have been shown to be associated with osteoclastogenesis and osteogenesis processes in OP. In our previous study, we discovered that miR-378 inhibits bone marrow mesenchymal stem cell (BMSC) osteogenesis and bone formation during fracture healing. However, the role of miR-378 during OP progression is not validated. In this study, we found that miR-378 transgenic (Tg) mice exhibited excessive bone loss after ovariectomy (OVX) treatment. MiR-378 increased BMSC’s osteoclastogenesis by activating both canonical and non-canonical nuclear factor kappa-light-chain-enhancer of activated B (NFκB) signaling pathway. Tumor necrosis factor receptor-associated factor 3 (Traf3) was directly regulated by miR-378 during osteoclast differentiation. miR-378 also aggravated transforming growth factor beta (TGFβ) impaired osteogenesis upon OVX treatment. Traf3 was involved in this process as well. In *in vivo* study, the intravenous injection of anti-miR-378 lentivirus could significantly rescue OVX induced bone loss and bone microarchitecture impairment. This study uncovered the novel role of miR-378 in OVX induced osteoporosis. The potential of developing miRNA-378 inhibitors as novel diagnostics or blockers as therapeutics for osteoporosis is worth exploring.

## Introduction

Bone remodeling is a dynamic equilibrated process which is tightly regulated by bone resorption mediated by osteoclasts and bone formation intervened by osteoblasts. Bone homeostasis of resorption and formation is achieved through the coordination of systematic and local factors such as hormones, hereditary regulators, and life conditions. Loss of bone homeostasis may lead to bone formation inhibition and bone resorption enhancement, which are factors leading to bone loss and further osteoporosis (OP).^1^ Osteoporosis is a degenerative disease characterized by decreased bone mass and damage to bone microarchitecture, as well as increased bone fragility majority occurring in women over the age of 50.^2^ The ideal therapeutic strategy of OP is to inhibit osteoclast generation which leads to bone resorption, while promoting bone formation.

MicroRNAs (miRNAs) are a class of small non-coding single-stranded RNA molecules of approximately 18–24 nucleotides in length encoded by endogenous genes and are involved in post-transcriptional gene expression regulation. Generally, miRNAs exert their regulatory functions by binding to the prime untranslated region (3′UTR) of mRNA to affect mRNA translation and degradation.^3^ Specifically, several miRNAs were revealed to possess dual regulating activities on both osteogenesis and osteoclastogenesis. MiR-20a has been shown to augment osteogenesis by simultaneously promoting bone marrow mesenchymal stromal cells (BMSCs) osteogenesis and suppressing bone resorption.^4^^,^^5^ Similarly, silencing miR-503 activated osteoclast formation of peripheral blood mononuclear cells and exaggerated the OP development in ovariectomized mice.^6^ Our group also found that miR-503 could accelerate BMSCs osteogenesis during femur distraction osteogenesis.^7^ The finding suggested the common dual regulating role of miRNA in regulating both osteoclastogenesis and osteogenesis, which make putative miRNAs as therapeutic targets for OP treatment.

MicroRNA-378 (miR-378) is a conserved miRNA that participates in multiple physiological and pathological processes of bone and cartilage remodeling. In our previous study, we have characterized that miR-378 could repress BMSCs osteogenesis and impede bone regeneration in the femur fracture model.^8^ However, the role and mechanism of miR-378 in osteoporosis remains speculative. The osteolytic metastasis study indicated that miR-378 expression was elevated during osteoclast differentiation, which suggested that miR-378 may also participate in the osteoclastogenesis process during OP.^9^ In this study, we aimed to analyze the function of miR-378 in OVX-induced bone loss from both osteoclastogenesis and osteogenesis perspectives. Our findings suggest that miR-378 overexpression significantly exaggerates ovariectomy (OVX)-induced bone loss. Furthermore, Tumor necrosis factor receptor-associated factor 3 (Traf3) was found to be the key factor involved in miR-378 promoted osteoclastogenesis as well as miR-378 elevated transforming growth factor beta (TGFβ) caused osteogenesis impairment.

## Materials & methods

### miR-378 TG mouse and ovariectomized osteoporosis model

The miR-378 transgenic (Tg) mice were obtained from Prof. Dahai Zhu’s lab (Chinese Academy of Medical Sciences).^10^ The miR-378 transgene was established based on C57BL/6J mice and globally overexpressed in miR-378 Tg mice under the control of the β-actin promoter (pCAGGS). The genotyping characterization of miR-378 TG mice was performed as described in our previous study.^8^ C57BL/6J female mice were used as WT control. All animal experiments were approved by the Ethics Committee of Chinese University of Hong Kong (19-229-MIS) and performed in accordance with the Code of Ethics of the World Medical Association. Sixteen of C57BL/6J and sixteen of miR-378 Tg female mice at the age of 3 months old were housed in separate ventilated cages for a 12 h light/dark cycle. Each type of mice was randomly divided into ovariectomy (OVX) and control group. The OVX mice were performed with ovariectomy by removing the bilateral ovaries as described previously,^11^ while the sham group received a sham operation without ovary dissection. All mice were subcutaneously injected with Calcein green (10 mg/kg) and xylenol orange (90 mg/kg) at days 14 or 4, respectively before euthanasia. Four weeks after surgery, the mice were terminated under anesthesia, and the femurs and serum from mice of each group were collected for further histological and immunohistochemical analyses. For the therapeutic experiment, the anti-miR-378 lentivirus was designed and constructed by GenePharma Company (GenePharma, China). Two groups of 3-month-old miR-378 Tg mice (*n* = 8 per group) were applied with OVX surgery. One week after surgery, one group of mice was treated with intravenous (IV) injection of lentivirus-incorporated miR-378 inhibitor with the lentivirus dose of 1x10^8^ particles resuspended in 5 μl PBS, and the other group was treated with corresponding control lentivirus once every week for 3 weeks. Mice were euthanized 3 weeks after the first lentivirus injection.

### Micro computed tomography

Micro computed tomography (micro-CT) analysis of distal femoral metaphysis (*n* = 8 per group) was analyzed using high-resolution μCT 40 (Scanco Medical, Bruttisellen, Switzerland). Image acquisition was performed at 70 kV and 118 μA, with an isotropic resolution of 12 μm per voxel. The segmentation parameters for bone from background were fixed at the threshold of 211 mg hydroxyapatite/cm^3^. For the measurement of trabecular bone, the volume of interest (VOI) inside the distal femurs was defined starting from the growth plate and proceeded at 200 continuous sections (10 μm/section) at proximal sides. Bone volume/tissue volume (BV/TV), trabecular number (Tb.N), trabecular thickness (Tb.Th), and bone mineral density (BMD) were determined and analyzed with a built-in program.

### Bone histomorphometric and histological analysis

The left distal femur (*n* = 8 per group) from each group was collected and fixed in 10% buffered formalin for 24 h, followed by dehydration and embedded in methyl methacrylate. The samples were then sectioned into 7-μm thickness the RM2155 hard tissue microtome (Leica, Wetzlar, Germany). The unstained sections were used for dynamic histomorphometric analysis. The sections were stained with von Kossa/nuclear fast red or toluidine blue for static histomorphometric measurements.^11^ The bone histomorphometric parameters including osteoblast surfaces per bone surface (Ob.S/BS), osteoclast surface per bone surface (Oc.S/BS) and mineral apposition rate (MAR) by using a semi-automatic digitizing image analysis system (OsteoMetrics, Atlanta, GA, USA) as mentioned previously.^12^ For histological studies, the femurs were fixed with 10% buffered formalin followed by 10% EDTA decalcification at 37 °C for 2 weeks. The tissues were then paraffin embedded and sectioned at 7-μm thickness and performed with TRAP staining (Sigma–Aldrich, St. Louis, USA) and IHC staining with primary rabbit anti-OCN (1:100, ab93876, Abcam, USA) antibody according to manufacturer’s instruction. The horseradish peroxidase-streptavidin system (Dako, Carpinteria, USA) was used for signal detection followed by counterstaining with hematoxylin.

### ELISA analysis

Four weeks after surgery, the blood was collected by retro-orbital puncture in each group and centrifuged to obtain serum. Serum levels of inflammatory factors were assessed using enzyme-linked immunosorbent assay (ELISA) kit detecting tumor necrosis factor ⟨ (TNF⟨, ABclonal Technology, Woburn, USA), interferon γ (IFNγ, Shanghai Jianglai Biological Technology, China) and tumor growth factor® (TGFβ, Shanghai Jianglai Biological Technology, China). The ELISAs were performed according to the instructions recommended by the manufacturers.

### BMNCs osteoclastogenesis

Primary bone marrow mononuclear cells (BMNCs) were isolated from femur bone marrow of WT and miR-378 Tg mice as previously described and were maintained in minimum essential medium alpha (⟨MEM) supplemented with 10% FBS. The BMNCs were firstly stimulated with M-CSF (30 ng/ml) for 3 days for osteoclast precursor formation, and followed by RANKL (50 ng/ml) treatment for 4–5 days for multinucleated osteoclasts induction. Total RNA was extracted for real-time PCR analysis. For tartrate-resistant acid phosphatase (TRAP) staining, cells were fixed in 4% paraformaldehyde for 10 min and then stained for Tartrate-resistant acid phosphatase (TRAP) activity according to the manufacturer’s instructions (Sigma–Aldrich, St. Louis, USA). The staining pattern of the cells was captured using a Leica DMIRB Inverted Leica Modulation Contrast Microscope (Leica, Wetzlar, Germany). The TRAP-positive multinucleated (nuclei>3) cells were scored as osteoclasts.

### BMSCs osteogenesis

BMSCs were isolated from bone marrow of WT and miR-378 Tg mice from both sham and OVX group at week 4 post OVX surgery.^8^ BMSCs at passage 3–4 were cultured in αMEM. For the study of TGFβ impairing effect, before osteogenesis induction, the BMSCs were pre-treated with TGFβ (1 ng/ml, 75362, Cell Signal Technology, Danvers, USA) for 3 days. After that, the osteo-induction was performed with the application of osteogenesis medium (Life Technologies, Gaithersburg, USA). Total RNA was extracted on day 7 post induction for real-time PCR analysis. Alkaline phosphatase (ALP) staining or Alizarin Red S (Sigma–Aldrich, St. Louis, USA) staining was also performed on day 7 or day 14 after osteogenic induction, respectively.^13^ For ALP activity assay, cells were PBS washed and stained with BCIP/NBT Color Development Substrate (Promega, Madison, USA), and incubated in the dark for 10 min at room temperature. The colorimetric reaction was terminated by washing with water. For Alizarin red S staining, BMSCs were PBS washed, 4% formaldehyde fixed, and stained with 40 mM Alizarin red S solution (Merck, Darmstadt, Germany). The culture plates were incubated at room temperature for 30 min with gentle shaking, washed twice, and air-dried. The ALP and ARS staining images were captured by using a Leica DMIRB Inverted Leica Modulation Contrast Microscope (Leica, Wetzlar, Germany). For ALP assay, the staining intensities were quantified by ImageJ software (NIH, Bethesda, USA). For ARS staining, the bound dyes were removed and dissolved in 10% cetylpyridinium chloride. The absorbance was measured at 560 nm using a microplate reader.

### Dual luciferase assay

Dual-luciferase assay was performed following the previous protocol.^14^ HEK293 cells were transfected with pmiRGLO-Traf3 wide type (wt) and site-mutation (mut) recombinant vectors together with miR-378-5p mimics. The Renilla luciferase vector was co-transfected as normalization control. The luciferase activities were measured using PerkinElmer VictorTM X2 2030 multilabel reader (Waltham, USA) and normalized with the Renilla luciferase activity.

### LNA GapmeR knockdown

Antisense LNA GapmeR against Traf3 was designed and produced by EXIQON (QIAGEN). BMNCs or BMSCs were transfected with 50 nM anti-Traf3 LNA GapmeR using Lipofectamine3000 reagent (Thermo Fisher, Waltham, USA) for 48 h, following the manufacturer’s recommended protocol. The knockdown efficiency was confirmed by Real-time PCR analysis.

### RNA extraction and real-time PCR

Cells were *in situ* lysed and total RNA was collected by using RNAiso (Takara, Kusatsu, Japan). Single-stranded cDNA was reverse transcribed from the RNA template using PrimeScript RT Master Mix (TaKaRa, Kusatsu, Japan). Quantitative real time-PCR was performed using Power SYBR Green PCR Master Mix (Thermo Fisher, Waltham, USA) on a QuantStudio 12K Flex Real-Time PCR system (Thermo Fisher, Waltham, USA). PCR amplifications were performed based on the manufacturers' protocol. The result normalization was performed using the 2–ΔΔCT method. Specific primers used are shown in Table S1.

### Western blot

The whole cell lysates were prepared from cultured cells using Radioimmunoprecipitation assay (RIPA) buffer (Sigma, St. Louis, USA) containing protease and phosphatase inhibitors (Roche, Mannheim, Germany). Fractionation of cytoplasmic and nuclear protein was performed using NE-PER Nuclear and Cytoplasmic Extraction Reagents (Pierce Biotechnology, Rockford, USA). Cytoplasmic and whole cell extracts (20 μg) and nuclear extracts (15 μg) were electrophoresed on 10% SDS-PAGE and transferred to polyvinylidene difluoride (PVDF) membrane (Bio-Rad, Hercules, USA). The membrane was blocked with 5% milk for 1 h and incubated with the primary antibodies including rabbit anti–NF–κB p65 (1: 3,000, ab16502, Abcam, Cambridge, UK), mouse anti-®-catenin (1:3,000, 610153, BD, Sparks, USA), mouse anti-®-actin (1:3,000, Sc-8432, Santa Cruz, USA), NF-κB p100/p52 (CST-4882, Cell signaling technology, Danvers, USA), NIK (CST-4994, Cell signaling technology, Danvers, USA), Rel B (CST-10544, Cell signaling technology, Danvers, USA), mouse anti-c-Fos (1:2,000, Ab208942, Abcam, Cambridge, UK), rabbit anti-NFATc1 (CST-8032S Cell signaling technology, Danvers, USA), rabbit anti-TRAF3 (1:3,000, A15106, Abclonal, Woburn, USA) and rabbit anti-Lamin B (1:3,000, CST-12586, Cell signaling technology, Danvers, USA) at 4 °C overnight. After 1-h incubation with horseradish-peroxidase (HRP)-conjugated secondary antibody (1:3,000, Thermo Fisher, Waltham, USA), the membrane was developed using the ECL system (Cell Signaling, Danvers, USA) and then exposed with X-ray film (Kodak, Rochester, USA). The films were scanned using Quantity One software (Bio-Rad, Hercules, USA). The original uncropped images of all Western blots are shown in Fig. S3.

### Immunofluorescence staining

The cells were fixed with 4% phosphate paraformaldehyde (Sigma–Aldrich, St. Louis, USA) solution and PBS washed. Cells were then blocked with 5% BSA and incubated with primary antibody (rabbit anti-β-catenin, 1:100, ab16051, Abcam, Cambridge, UK) in BSA overnight at 4 °C. Subsequently, cells were incubated with the fluorophore-conjugated goat anti-rabbit IgG-H&L (Alexa Fluor® 488) (1:100, ab150077, Abcam, Cambridge, UK) for 30 min. The cells were counterstained with DAPI. Photographs of selected areas were taken under a Leica DMIRB Inverted Leica Modulation Contrast Microscope (Leica, Wetzlar, Germany).

### Statistical analysis

All data were presented as median and interquartile range or mean ± medians (minimum–maximum) according to graph types applied. Experiments were repeated independently at least three times, and representative data are shown. The statistical significance among multiple groups was assessed using One-way ANOVA for comparisons, followed by Tukey’s Honestly Significant Difference (HSD) test for post-hoc analysis. The statistical significance between two independent groups was evaluated using Two-tailed student’s *t*-test with Welsh’s correction. The analysis was performed with GraphPad Prism 8 (GraphPad Software, USA). *P* < 0.05 was revealed as a significant difference.

## Results

### miR-378 overexpression exaggerated ovariectomy induced bone loss

**Figure 1:**
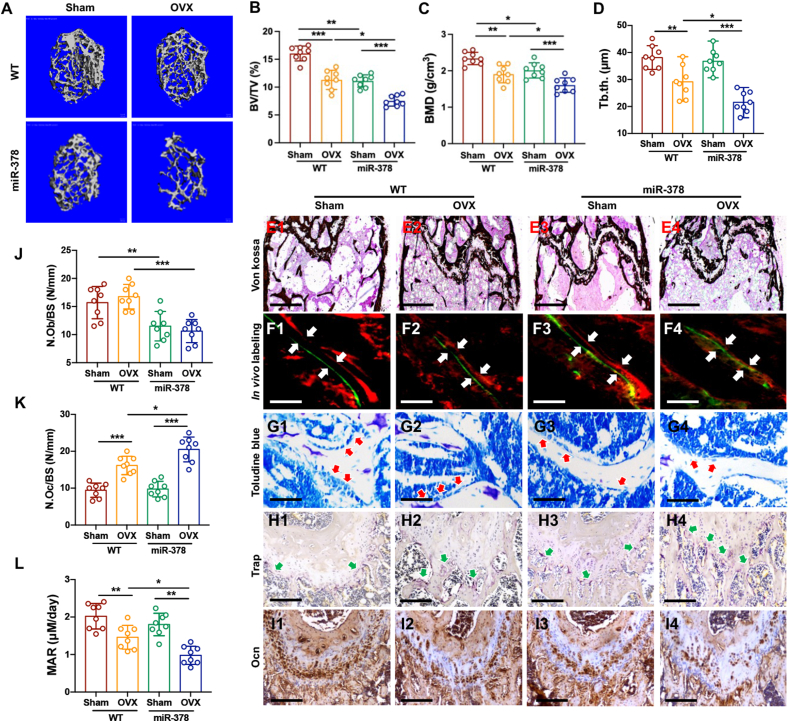
miR-378 overexpression accelerated bone loss upon ovariectomy. **(A)** Three-dimensional reconstruction of trabecular bone in the distal metaphysis of WT and miR-378 TG mice after sham and OVX treatment. Scale bar, 100 μM. **(B–D)** Microstructure parameters including bone volume/tissue volume (BV/TV) (B), bone mineral density (BMD) (C) and trabecular thickness (Tb.Th) (D) of the trabecular bone in different groups. **(E–I)** Representative images of Von Kossa staining (E), *in vivo* double labels (F) and Toluidine blue staining (G) of femoral metaphysis as well as IHC staining using TRAP (H) and OCN (I) antibodies. White arrow indicates the bone distance between two labeling which reflects the mineral apposition rate. Red arrow indicates the osteoblasts. Green arrow indicated the TRAP positive cells. Scale bar: 400 μM for Von Kossa, 100 μM for *in vivo* labeling and Toluidine blue staining, 200 μM for IHC staining. **(J–L)** Histomorphometric analysis of distal femur sections including N.Ob/BS (number of osteoblasts per bone surface) (J), N.Oc/BS (number of osteoclast per bone surface) (K) and MAR (mineral apposition rate) (L) (*n* = 8; ∗*p* < 0.05, ∗∗*p* < 0.01, ∗∗∗*p* < 0.001).

Three-dimensional reconstruction of trabecular bone at distal metaphysis was compared between WT and miR-378 Tg mice in both sham and OVX groups. The bone architecture of the OVX group was significantly impaired compared with that of the WT group. MiR-378 overexpression further exaggerated the OVX induced bone loss (Fig. 1A). The bone microstructure parameter analysis also revealed that OVX decreased BV/TV, BMD and Tb.Th. levels were more severe in miR-378 Tg mice compared with WT control (Fig. 1B–D). The miR-378 Tg mice femur also showed decreased calcium deposit and lower bone formation rate upon OVX treatment as revealed by Von Kossa staining, *in vivo* double labeling and toluidine blue staining, respectively (Fig. 1E–G). Histomorphometric analysis of distal femur sections showed that OVX treatment reduced N.Ob/BS and MAR, while increased N.Oc/BS in the distal metaphysis of trabecular bone. The specific expression patterns were further aggregated by miR-378 overexpression (Fig. 1J–L). The histological staining result indicated that the osteoclast amount in the trabecular bone surfaces was obviously increased in the distal head of femurs in miR-378 mice upon OVX surgery, as revealed by TRAP-positive staining area (Fig. 1H, red arrows). While the expression of osteocalcin (OCN), an osteogenesis indicator was reduced in the OVX group compared to the Sham group, and this decrease was intensified by miR-378 overexpression (Fig. 1I).

### miR-378 accelerated osteoclast differentiation upon RANKL stimulation

**Figure 2:**
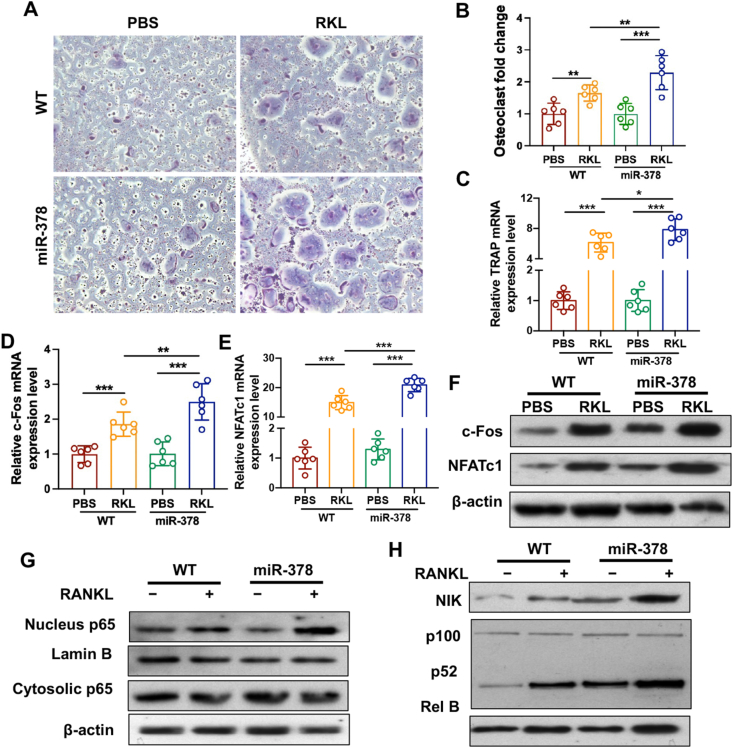
miR-378 increased osteoclast differentiation via activating both canonical and non-canonical NF-κB signaling pathway. **(A)** TRAP staining assay of BMMs isolated from WT and miR-378 TG mice at day 5 after RANKL-induced osteoclastogenesis was performed. Scale bar: 1000 μM. **(B)** Quantitative data of osteoclast fold change (*n* = 6; ∗*p* < 0.05, ∗∗*p* < 0.01). **(C–E)** The mRNA expression level of osteoclastogenic differentiation related markers including TRAP(C), c-Fos (D) and NFATc1 (E) measured by qRT-PCR (*n* = 6; ∗*p* < 0.05, ∗∗*p* < 0.01, ∗∗∗*p* < 0.001). **(F)** Western blot analysis of c-Fos and NFATc1 expression level in different groups. **(G, H)** Western blot analysis of NF-κB/p65 nucleus translocation (G) as well as NIK, p100/p52 and Rel B protein levels (H) in BMNCs after treated with RANKL for 5 days.

To study the effect of miR-378 overexpression on BMNCs osteoclastogenesis activity, BMNCs were isolated from WT and miR-378 mice and osteoclastogenic induction was performed. Our result suggested that after RANKL induction, miR-378 expression level was further increased in miR-378 BMNCs compared with WT BMNCs (Fig. S1). The TRAP staining was performed and the result indicated that BMNCs isolated from miR-378 mice showed a higher osteoclast differentiation ability compared with that of the WT mice (Fig. 2A). The semi-quantitative analysis of osteoclast fold change showed that miR-378 overexpression increased osteoclast formation by nearly 50% compared with the WT group upon RANKL treatment (Fig. 2B). BMNCs from miR-378 Tg mice showed higher mRNA expression level of osteoclast differentiation, fusion and activity markers including TRAP, c-Fos, NFATc1, DC-STAMP, TRAF6, c-Src, Ctsk and Vav3 than the WT group after osteoclastogenic induction (Fig. 2C–E; Fig. S2). Western blot analysis results also revealed higher c-Fos and NFATc1 protein expression in miR-378 BMNCs after RANKL-induced osteoclast differentiation (Fig. 2F). NF-κB signaling pathways, both canonical and non-canonical, were largely involved in the process of osteoclast differentiation. To study whether miR-378 regulated osteoclastogenesis via mediating NF-κB signaling, cytosolic and nucleus NF-κB/p65, p100, p52 as well as NIK and Rel B protein levels in BMNCs were analyzed (Fig. 2G, H). Upon RANKL induction, miR-378 significantly increased the p65 nucleus translocation, while it activated more vigorous p100/p52 processing as well as NIK and Rel B upregulation. The result suggested that miR-378 increased osteoclast differentiation activity mainly by activating non-canonical rather than canonical NF-κB signaling pathway.

### Traf3 was directly regulated by miR-378 during osteoclast differentiation

**Figure 3:**
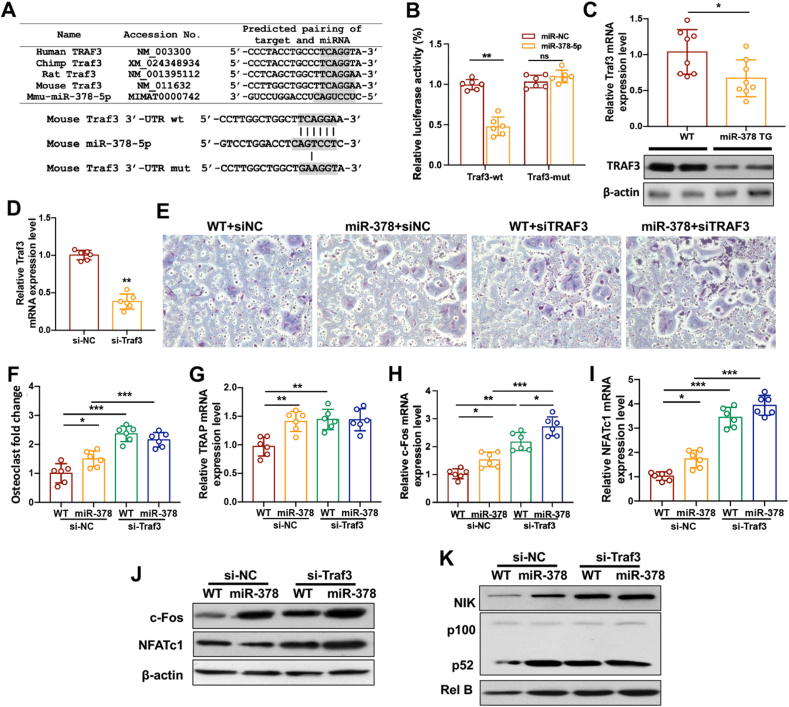
Traf3 was directly regulated by miR-378 during osteoclast differentiation. **(A)** Conservation of the miR-378-5p binding site on Traf3 3′-UTR (shaded region) among different species. The wild type (wt) and mutation (mut) form of Traf3 3′-UTR luciferase reporter vector were shown. **(B)** Effects of miR-378-5p on the luciferase activity of pmiRGLO vectors incorporated with Traf3-wt or Traf3-mut sequence were measured. **(C)** The mRNA and protein expression level of Traf3 in WT and miR-378 BMNCs were measured respectively. **(D)** Traf3 LNA knockdown efficiency in BMNCs was measured. **(E)** BMNCs from WT and miR-378 Tg mice were Traf3 knockdown and the TRAP staining assay was performed at day 5 after RANKL-induced osteoclastogenesis. Scale bar: 1000 μM. **(F)** Quantitative data of osteoclast fold change. **(G–I)** The mRNA expression level of osteoclastogenesis related markers including TRAP (G), c-Fos (H) and NFATc1 (I) measured by real-time PCR (*n* = 6; ∗*p* < 0.05, ∗∗*p* < 0.01, ∗∗∗*p* < 0.001). **(J)** Western blot analysis of c-Fos and NFATc1 expression level in different groups. **(K)** Western blot analysis of NIK, p100/p52 and Rel B protein levels in BMNCs after cells treated with RANKL for 5 days.

To identify the putative miR-378 target genes which may possess the regulating effect on both canonical and non-canonical NF-κB signaling activities, bioinformatics analysis was applied by using Targetscan software (www.targetscan.org). *In silico* analysis results revealed that Traf3 was one of the direct target genes for miR-378-5p. Specifically, Traf3 was involved in both canonical and non-canonical NF-κB signaling pathways. Traf3 was also reported to limit osteoclast formation in inflammatory diseases. All these results indicated that miR-378 probably regulates osteoclastogenesis in a Traf3 dependent manner. The conservation of the miR-378-5p binding site on the Traf3 3′-UTR in different species was compared. The wild type (wt) and mutation (mt) of the Traf3 3′-UTR luciferase reporter vectors were constructed based on the predicted binding sequence (Fig. 3A). miR-378-5p could significantly reduce the luciferase activity of pmiRGLO vectors incorporating Traf3-wt sequence. However, this luciferase activity reduction was not revealed in Traf3-mut groups (Fig. 3B). The miR-378 Tg BMNCs also showed reduced Traf3 mRNA and protein expression (Fig. 3C). To study the mediation effect of Traf3 in miR-378 downregulated osteoclastogenesis, Traf3 expression was successfully abolished upon LNA knockdown (Fig. 3D). The TRAP staining assay at day 5 after RANKL-induced osteoclastogenesis indicated that miR-378 Tg BMNCs showed a higher osteoclastogenesis activity compared with the WT group. However, the Traf3 knockdown abolished this difference, which suggested that miR-378 activates osteoclastogenesis via inhibiting Traf3 expression (Fig. 3E, F). The mRNA and protein expression levels of osteoclastogenesis-related markers including TRAP, c-Fos and NFATc1 were measured by real-time PCR and Western blot analysis. All the three markers were significantly elevated in miR-378 Tg group compared with WT group with siNC treatment. Knockdown of Traf3 even increased the expression of these markers. However, the differences between WT and miR-378 Tg group were diminished for c-Fos and NFATc1, but not for TRAP (Fig. 3G–I). Western blot result patterns also confirmed the regulatory role of Traf3 in miR-378 mediated osteoclastogenesis process (Fig. 3J). The non-canonical NF-κB signaling pathway has also been shown to be involved in Traf3-manipulated osteoclastogenesis. NIK, p52 and Rel B protein expression were increased by miR-378 and significantly promoted by Traf3 knockdown (Fig. 3K). Thus, miR-378 promotes osteoclastogenesis in a Traf3 dependent manner and Traf3 knockdown may result in severe osteoclastogenesis.

### miR-378 was involved in TGFβ-impaired osteogenesis during ovariectomy

**Figure 4:**
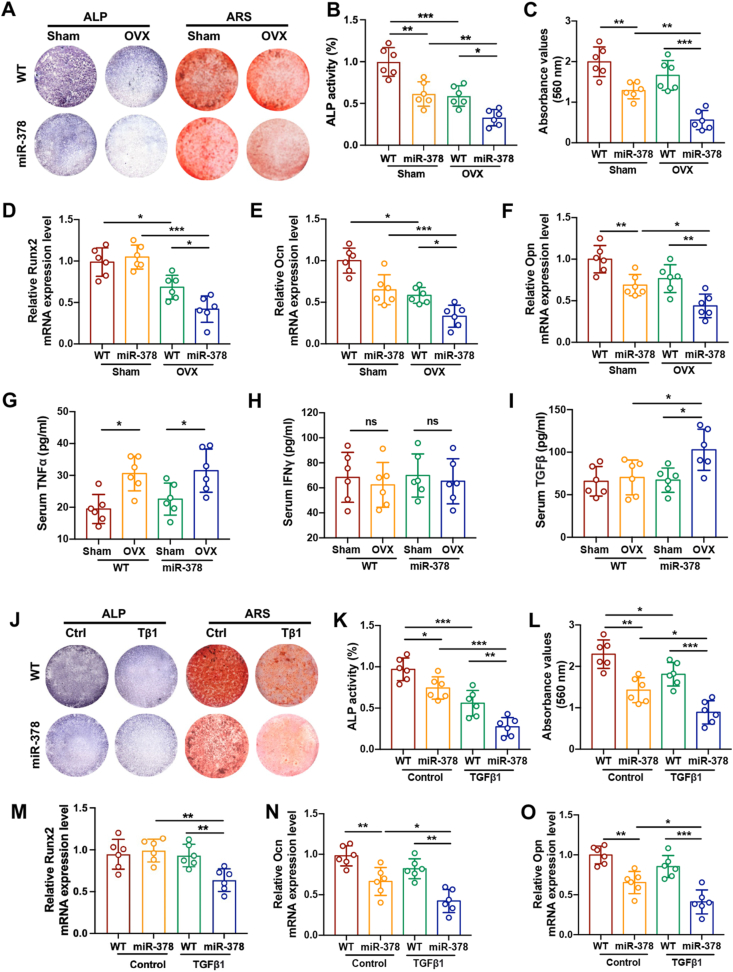
miR-378 was involved in TGFβ impaired osteogenesis in osteoporotic mice. **(A)** ALP staining and Alizarin Red S staining of BMSCs from WT and miR-378 Tg mice upon sham and OVX operations, respectively and osteogenic induced for 7 days. Scale bar: 10 mm. **(B, C)** Quantitative analysis of ALP activities (B) and ARS staining intensities (C). **(D−F)** The mRNA expression level of osteogenesis markers including Runx2 (D), Ocn (E) and Opn (F) measured by real-time PCR. **(G−I)** Total TNF⟨ (G), IFN© (H) and TGFβ (I) serum levels in WT and miR-378 Tg mice after sham and OVX surgery. **(J)** ALP staining and Alizarin Red S staining of BMSCs from WT and miR-378 Tg mice pretreated with TGFβ. Scale bar: 10 mm. **(K, L)** Quantitative analysis of ALP activities (K) and ARS staining intensities (L). **(M−O)** The mRNA expression level of osteogenesis markers including Runx2 (M), Ocn (N) and Opn (O) measured by real-time PCR (*n* = 6; ∗*p* < 0.05, ∗∗*p* < 0.01, ∗∗∗*p* < 0.001).

To study the BMSCs osteogenesis activity of miR-378 Tg mice under osteoporotic conditions, the BMSCs were isolated from both WT and miR-378 Tg mice in sham and OVX group, and performed with osteogenic induction. The ALP and Alizarin Red S staining result revealed that BMSCs isolated from OVX mice showed a lower ALP activity and decreased calcium deposition, which reflects a weaker osteogenesis activity compared with BMSCs from sham group, while miR-378 overexpression further aggravated this decrease (Fig. 4A–C). The mRNA expression levels of osteogenesis markers including Runx2, Ocn and Opn also displayed the same pattern. The expression of BMSCs osteogenesis markers were downregulated upon OVX treatment and much worsened by miR-378 overexpression (Fig. 4D–F). Further studies were conducted to discover the underlying mechanism by which miR-378 severely impairs BMSCS osteogenesis under osteoporotic conditions. Previous study uncovered that several pro-inflammatory cytokines were the association between inflammation and osteoporosis. Certain inflammatory conditions could further limit the BMSCs osteogenesis. In our study, the serum level of certain inflammatory factors, including TNF⟨, IFN© and TGFβ were analyzed in WT and miR-378 Tg mice with/without OVX surgery (Fig. 4G–I). Among them, TGFβ serum level was significantly upregulated in miR-378 Tg mice after OVX treatment. The findings indicated that TGFβ is probably the key factor which mediates the impairment of osteogenesis induced by miR-378 overexpression. The ALP staining and Alizarin Red S staining of BMSCs after osteo-induction revealed that miR-378 downregulated BMSCs osteogenic activity, while pre-treatment of TGFβ further worsened it (Fig. 4J−L). Analysis of mRNA expression level of osteogenesis markers including Runx2, Ocn and Opn further strengthened our conclusion that the inhibitory effect of miR-378 on osteogenic capacity of BMSCs could be exaggerated by TGFβ treatment (Fig. 4M−O).

### miR-378 mediated TGFβ-impaired osteogenesis by downregulating Traf3/β-catenin signaling

**Figure 5:**
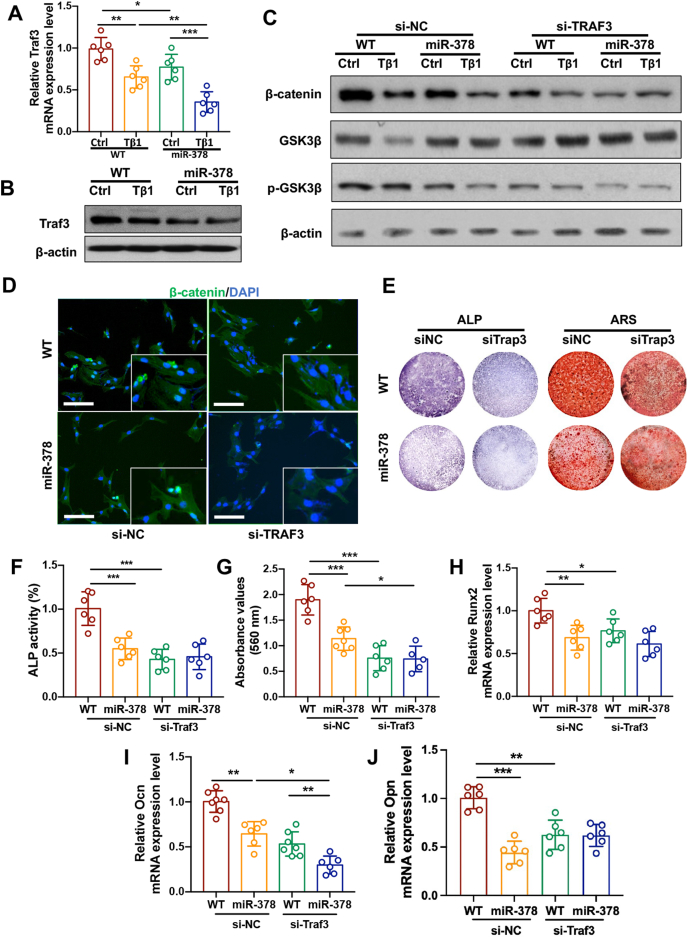
miR-378 mediated TGFβ-induced osteogenesis via downregulating Traf3/β-catenin signaling. **(A, B)** Traf3 mRNA and protein expression level in WT and miR-378 Tg BMSCs treated with TGFβ. **(C)** Western blot analysis of β-catenin expression and GSK3β in WT and miR-378 Tg BMSCs after Traf3 knockdown and TGFβ treatment. **(D)** Immunofluorescence staining of β-catenin expression in WT and miR-378 Tg BMSCs upon si-Traf3 transfection and TGFβ treatment. Scale bar: 100 μm. **(E−G)** ALP staining and Alizarin Red S staining (E) and quantitative analysis of ALP activities (F) and ARS staining intensities (G) of WT and miR-378 Tg BMSCs mice upon si-Traf3 transfection and osteogenic induced for 7 days. Scale bar: 10 mm**. (H−J)** The mRNA expression level of osteogenesis markers including Runx2 (H), Ocn (I) and Opn (J) measured by real-time PCR (*n* = 6; ∗*p* < 0.05, ∗∗*p* < 0.01, ∗∗∗*p* < 0.001).

Previous study suggested that Traf3/β-catenin signaling pathway was involved in TGFβ impaired osteogenesis.^15^ In our study, we found that Traf3 expression level in BMSCs was downregulated by TGFβ treatment and this repression was further strengthened by miR-378 overexpression (Fig. 5A, B). Western blot analysis results also revealed that β-catenin expression and GSK3β phosphorylation in BMSCs were synergistically downregulated by both miR-378 overexpression and TGFβ stimulation. While this synergistic effect was abolished by Traf3 knockdown (Fig. 5C). The mediating role of Traf3 on miR-378 repressed β-catenin signaling was further demonstrated by immunofluorescence (IF) staining of β-catenin expression in WT and miR-378 Tg BMSCs after si-Traf3 treatment (Fig. 5D). ALP staining and Alizarin Red S staining as well as quantitative analysis indicated that miR-378 exerted its osteogenic inhibitory effect by repressing Traf3 expression (Fig. 5E–G). The mRNA expression pattern of osteogenic markers Runx2, Ocn and Opn also indicated that miR-378 exerts its inhibitory effect on osteogenesis while being alleviated by Traf3 knockdown (Fig. 5H–J). The study result indicated that miR-378 overexpression elevated TGFβ impairs BMSCs osteogenesis via downregulating Wnt/β-catenin signaling in a Traf3 dependent manner.

### Anti-miR-378 lentivirus injection rescued ovariectomy induced bone loss in miR-378 Tg mice

**Figure 6:**
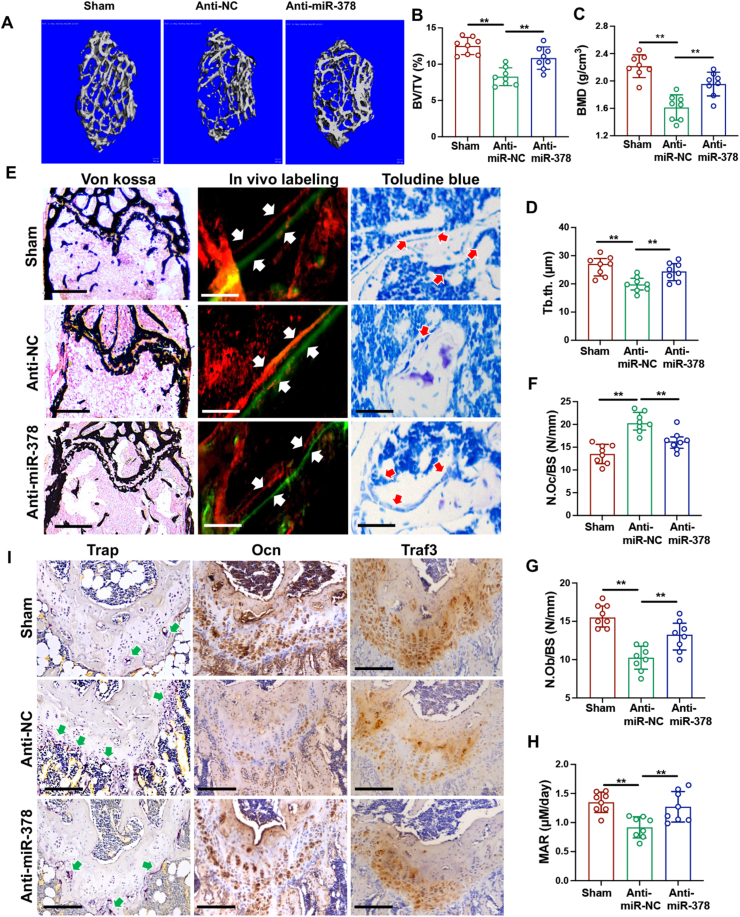
Anti-miR-378 lentivirus injection rescued ovariectomy induced bone loss in miR-378 Tg mice. **(A)** Three-dimensional reconstruction of trabecular bone in the distal metaphysis of miR-378 TG mice after anti-NC and anti-miR-378 mediated lentivirus injection. Scale bar, 100 μM. **(B−D)** Microstructure parameters including bone volume/tissue volume (BV/TV) (B), bone mineral density (BMD) (C) and trabecular thickness (Tb.Th) (D) of the trabecular bone in different groups. **(E)** Representative images of Von Kossa staining, *in vivo* double labels and Toluidine blue staining of femoral metaphysis. White arrow indicates the bone distance between two labeling which reflects the mineral apposition rate. Red arrow indicates the osteoblasts. Scale bar: 400 μM for Von Kossa, 100 μM for *in vivo* labeling and Toluidine blue staining. **(F–H)** Histomorphometric analysis of distal femur sections including N.Ob/BS (G), N.Oc/BS (H) and MAR (I) (*n* = 8; ∗*p* < 0.05, ∗∗*p* < 0.01, ∗∗∗*p* < 0.001). **(I)** TRAP staining and IHC staining of femoral metaphysis using Ocn and Traf3 antibodies. Red arrow indicates the TRAP positive cells. Scale bar: 200 μM.

Due to the significant effect of miR-378 on promoting osteoclastogenesis while inhibiting osteogenesis during osteoporosis, the therapeutic effect of anti-miR-378 lentivirus upon tail vein injection on OVX-induced bone loss was performed to test its therapeutic potential. Three-dimensional reconstruction of trabecular bone in the distal metaphysis indicated that the bone mass loss and microarchitecture impair in OVX miR-378 Tg mice were significantly rescued upon anti-miR-378 mediated lentivirus injection but still lower than the level of sham group (Fig. 6A). Analysis of microstructure parameters of the trabecular bone including BV/TV, BMD and Tb.Th. further demonstrated the therapeutic effectiveness of anti-miR-378 lentivirus treatment on OVX impaired bone quality (Fig. 6B–D). Representative images of von Kossa staining, *in vivo* double labeling and toluidine blue staining of distal femoral metaphysis exhibited the improved bone microarchitecture and bone growth rate after anti-miR-378 treatment (Fig. 6E). Histomorphometric analysis of distal femur sections including N.Ob/BS, N.Oc/BS and MAR demonstrated the promoting effect of osteogenesis while the inhibiting effect of osteoclastogenesis of anti-miR-378 during osteoporosis process (Fig. 6F–H). IHC staining results also revealed decreased TRAP staining and increased Ocn expression, as well as elevated expression of miR-378 targeting Traf3, upon anti-miR-378 treatment (Fig. 6I).

## Discussion

MiRNAs have been identified as the key roles in bone metabolism, including osteocyte differentiation, osteoclastogenesis and osteogenesis. Recent studies have shown that miRNAs may serve as potential therapeutics for bone pathologies, including OP, osteonecrosis and osteoarthritis. In this study, we found that miR-378 Tg mice showed an accelerated OVX-induced bone loss. While miR-378 could facilitate all the steps of the osteoclast mature process including differentiation, fusion and activity. Then, we found that miR-378 significantly promotes osteoclastogenesis of BMNCs by activating canonical and non-canonical NF-κB signaling pathways. Traf3 was found to be the miR-378 direct target gene involved in this regulating process. In addition, we found that serum TGFβ level was elevated in miR-378 Tg mice upon OVX treatment, while Traf3 also mediated TGFβ impaired osteogenesis via Traf3/®-catenin signaling pathway. At last, we validated the therapeutic effect of anti-miR-378 in an OVX-induced OP mice model.

MiRNA has been demonstrated as effective regulators for multiple signaling pathways. MiRNAs were reported to be involved in both canonical and non-canonical NF-κB signaling during osteoclastogenesis.^16^ Canonical NF-κB pathway relies on the phosphorylation and degradation of IκBs, particularly IκBα, leading to nuclear translocation of the p50/p65 NF-κB complex. The phosphorylation of IκBα is mediated by the IκB kinase (IKK), which is a heterotrimer complex composed of IKKα and IKKβ and IKKγ.^17^ Besides, an alternative non-canonical NF-κB signaling pathway exists, which depends on the activation of NF-κB-inducing kinase (NIK) and the subsequent NF-κB p100 phosphorylation and processing into p52, leading to the nucleus translocation and activation of the RelB/p52 NF-κB complex.^18^ Studies have proved that both canonical and non-canonical NF-κB signaling pathways were widely involved in proliferation, differentiation and function of osteoblasts, osteoclast, and osteocytes.^19^ MiRNAs were largely involved in canonical NF-κB signaling regulated osteoclastogenesis. MiR-143 could inhibit osteoclast formation via repressing the canonical NF-κB signaling pathway, as revealed by ablation of RANK, NF-κB p65, and IκBα expression.^20^ MiR-128 also directly targeted SIRT1 and hindered its repressive effect on NF-κB p65 via lysine 310 acetylation, which further promoted osteoclastogenesis.^21^ In contrast, several miRNAs, including miR-223, miR-15a and miR-16 specifically modulated the non-canonical NF-κB signaling pathway by regulating the NIK stabilization expression during osteoclast differentiation.^22^ We found that both canonical and non-canonical signaling pathways were activated during osteoclastogenesis upon miR-378 overexpression, which inspired us to further characterize the putative miR-378 target gene which may be involved in both of these two distinct NF-κB regulating cascades.

In our study, we identified Traf3 as the putative genes directly targeted by miR-378. Traf3 mediated miR-378 elevated osteoclast differentiation via both canonical and non-canonical NF-κB signaling pathways. Osteoclasts differentiation and activation are mainly regulated by macrophage colony stimulating factor (MCSF) and receptor activator of nuclear factor-κB ligand (RANKL). MCSF initiates the differentiation of osteoclast precursors and induces the expression of RANK. RANKL from accessory cells in bone marrow could bind with RANK, activate the TNF receptor-associated factor 6 (TRAF6) and induce the activation of NF-κB in the osteoclastogenesis process. While another member of TRAF family, TRAF3 could form the heterodimer with TRAF2 and promote the degradation of NIK to limit the RANKL-induced non-canonical NF-κB signaling activity.^23^ TRAF3 could also attenuate IκBα and NF-kB p65 phosphorylation via recruiting and activating lymphotoxin β receptor (LTBR) signaling complexes, which also suggests the involvement of TRAF3 in canonical NF-κB signaling.^24^ In our study, Traf3 was found to be the pivotal factor in miR-378-mediated osteoclast differentiation. By associating with and downregulating Traf3, miR-378 plays a promoting role in osteoclast differentiation of BMNCs. Our study also suggested that non-canonical NF-κB activation by miR-378 in BMNCs was also abolished upon Traf3 knockdown. Other studies also discovered that osteoclast specific miR-214 contributes to osteolytic bone metastasis via binding Traf3 and repressing downstream NIK and NF-κB p65 expression.^25^ MiR-346-3p also directly regulated Traf3 expression and promoted osteoclastogenesis.^26^ These studies further demonstrated that Traf3 was an extensive target for miRNA on regulation of signaling during osteoclast formation.

In *in vivo* and *in vitro* study, we found that serum TGFβ was elevated in miR-378 Tg mice, and miR-378 mediated TGFβ impaired osteogenesis. Emerging evidence suggests that inflammatory factors played a significant role in bone diseases. Numerous pro-inflammatory factors have been implicated in the regulation of bone remodeling by mediating the function of osteoblasts and osteoclasts, which makes them important risk factors in bone disorder, including osteoporosis. Serum level of inflammatory factors, such as INF© and TNFα were significantly elevated in osteoporotic patients, which is associated with low bone mass and therefore an independent risk factor for osteoporosis.^27^ Specifically, miRNA was speculated to play a mutual role with inflammatory factors during OP. Tang et al discovered the similar expression pattern of TNFα and miR-144 in the serum of the postmenopausal OP group.^28^ Suarjana’s analysis revealed that miR-21 was positively correlated with serum RANKL level but negatively correlated with BMD in OP patients.^29^ In this study, we found that TGFβ serum level was significantly elevated in miR-378 Tg mice. The anti-osteogenic capacity of miR-378 was upregulated upon TGFβ treatment, which indicated a positive feedback loop between these two factors. In a previous study, TGFβ was reported to recruit perivascular MSCs and promote their differentiation in a Smad3 signaling dependent manner. However, the regulatory role of TGFβ on osteoblasts lifecycle is distinctive, promoting osteoblasts proliferation in the early stage while inhibiting terminal differentiation. Besides, TGFβ also exerts its regulatory functions on other bone cells. TGFβ promoted the migration of osteoclast precursors and activated the osteoclastogenesis process.^30^ TGFβ also manipulated osteocytes function by regulating osteocyte MMP13 expression.^31^ MiR-378 overexpression profoundly changes the bone homeostasis by promoting osteoclastogenesis while inhibiting osteogenesis. Bonewald’s group found the promoted osteoclast could release and activate bone matrix-bound TGFβ in a TGFβ-binding protein-1 (LTBP1)-related mechanism.^32^ Upon release, TGFβ elevation could further exuberate BMSCs osteogenesis during ovariectomy. TGFβ was identified as the corresponding regulator, which acted as a significant mediator of miR-378 impaired osteogenesis in osteoporotic mice.

Interestingly, we identified that Traf3, which was involved in miR-378 activated osteoclastogenesis, also participated in TGFβ impaired osteogenesis via Traf3/β-catenin signaling pathway. TRAF3 protein levels were reported to decrease in bone and bone marrow during aging in mice and humans. Traf3 MSC-specific knockout mice also developed early osteoporosis due to promoted osteoclast formation and reduced osteogenesis.^33^ Similar to our study, Boyce’s research found that Traf3 prevents GSK3β-mediated β-catenin degradation in BMSCs and maintain osteoblast formation, while Traf3 was degraded and Traf3 serum level was downregulated by TGFβ released from resorbing bone.^15^ The study result corresponds to our conclusions that miR-378 overexpression induced the downregulation of Traf3 expression and β-catenin signaling activity in BMSCs in a direct association and TGFβ-involved indirect way. The presence of miR-378 elevated the serum level of TGFβ, which subsequently promotes the degradation of Traf3 and thereby inhibits osteogenesis, as well as promotes osteoclastogenesis.

Based on the study of miR-378 regulation effect on both osteoclastogenesis and osteogenesis under osteoporotic condition, we further studied the therapeutic effect of anti-miR-378 mediated lentivirus on OP. The result indicated that the tail vein injection of anti-miR-378 lentivirus significantly rescued OVX-induced bone loss. Due to the precise targeting and regulation mechanism, miRNA mediated gene therapy has been revealed as a clinically applicable strategy for OP. Local subcutaneous injection of lentivirus incorporating miR-429 could accelerate the bone formation remodeling during fracture repair.^26^ Lentivirus-mediated miR-29 precursor tail vein injection also rescued the glucocorticoid induced bone loss as well as the disturbance of Wnt and DKK signaling in a rat model.^34^ In our study, we demonstrated the therapeutic effect of anti-miR-378 treatment on OVX-induced bone deterioration in miR-378 Tg mice. In our previous study, we also proved that anti-miR-378 mediated lentivirus injection could ameliorate cartilage degeneration in a surgery induced OA mice model. The treatment effectiveness of this miRNA inhibitor inspired us to consider that the anti-miR-378 lentivirus injection may have benefits on osteoarthritis complicated with subchondral bone deterioration. However, our study also has limitations. In our study, we only confirmed the amelioration effect of anti-miR-378 lentivirus on bone deterioration in miR-378 Tg mice. The potential therapeutic effect of anti-miR378 after OVX in WT mice should be further performed to confirm the potential of miR-378 as a therapeutic target.

In general, we found that miR-378 overexpression accelerated OVX-induced bone loss by regulating osteoclastic and osteogenic processes. MiR-378 promoted osteoclast differentiation by activating both canonical and non-canonical NF-κB signaling pathways. MiR-378 was also involved in TGFβ impaired osteogenesis during ovariectomy. Traf3 played as a mediating factor in both miR-378-regulated osteoclastogenic and osteogenic processes. The treatment effect of anti-miR-378 lentivirus injection significantly suggested to us that miR-378 could be developed as a prognosis and therapeutic target in OP treatment.

## CRediT authorship contribution statement

**Lu Feng:** Investigation, Conceptualization, Methodology. **Zhengmeng Yang:** Investigation. **Nan Hou:** Investigation. **Haixing Wang:** Investigation. **Shanshan Bai:** Investigation. **Xuan Lu:** Investigation. **Yaofeng Wang:** Validation. **Sien Lin:** Resources. **Micky D. Tortorella:** Supervision. **Gang Li:** Supervision.

## Conflict of interests

The authors declared no conflict of interests.
