# Supplementary material for: MiR-378 mediates the ovariectomy induced bone loss via exaggerating osteoclastogenesis and transforming growth factor beta impaired osteogenesis
Source: Genes Dis. 2025 Jul 3;13(1):101754. doi: 10.1016/j.gendis.2025.101754 (PMC12555769; doi:10.1016/j.gendis.2025.101754)
Supplement: Multimedia component 1 [file mmc1.docx]

**Table S1** Primers used for vector construction and Real-time PCR assays

| **Primers for Real-time PCR** | | | | |
| --- | --- | --- | --- | --- |
| Gene ID | Accession No. | F/R | Sequence | Product size (bp) |
| mRunx2 | NM_001146038 | F  R | ACACCGTGTCAGCAAAGC  GCTCACGTCGCTCATCTTG | 99 |
| mOcn | NM_007541 | F  R | GGTAGTGAACAGACTCCGGC  CAAGCAGGGTTAAGCTCACA | 96 |
| mOpn | NM_001204201 | F  R | ATTTGCTTTTGCCTGTTTGG  TGGCTATAGGATCTGGGTGC | 109 |
| mTRAP | NM_001102405 | F  R | CTGGAGTGCACGATGCCAGCGACA  TCCGTGCTCGGCGATGGACCAGA | 419 |
| mc-Fos | NM_010234 | F  R | CCAGTCAAGAGCATCAGCAA  AAGTAGTGCAGCCCGGAGTA | 247 |
| mNFATc1 | NM_016791 | F  R | CCGTTGCTTCCAGAAAATAACA  TGTGGGATGTGAACTCGGAA | 152 |
| mTraf3 | NM_011632 | F  R | TGAGCTGGAGAGCGTAGACA  AGATCAGCACCCCGTTGTAG | 173 |
| mDC-STAMP | NM_001289506 | F  R | TTGCCGCTGTGGACTATCTG  GAATGCAGCTCGGTTCAAAC | 168 |
| mTraf6 | NM_009424 | F  R | AACTGTGCTGTGTCCATGGC  CAGTCTCATGTGCAACTGGG | 246 |
| mSrc | NM_009271 | F  R | CAATGCCAAGGGCCTAAATGT  TGTTTGGAGTAGTAAGCCACGA | 123 |
| mCtsk | NM_007802 | F  R | GAAGAAGACTCACCAGAAGCAG  TCCAGGTTATGGGCAGAGATT | 102 |
| mVav3 | NM_020505 | F  R | TTAGGAACTACACTGGCACC  TTCTCCAGATTCTTTGGTCC | 317 |
| mGAPDH | NM_001289726 | F  R | CGTCCCGTAGACAAAATGGT  TTGATGGCAACAATCTCCAC | 110 |

“m” stands for “mouse”.


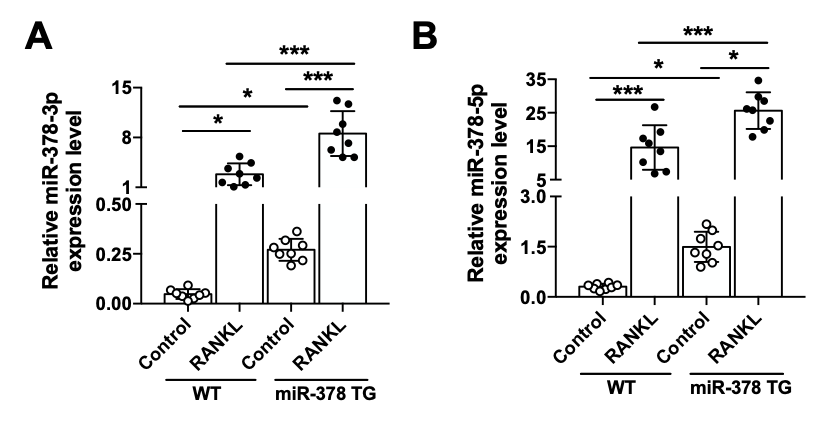


**Supplementary Figure 1. miR-378 expression level in BMNCs**

**A&B.** Expression level of miR-378-3p and **(A)** miR-378-5p **(B)** in BMNCs isolated from WT and miR-378 TG mice with/without RANKL induction were studied by using Real-time PCR. The relative miRNA expression was normalized to mmu-miR-103-3p (n=8; *p<0.05, **p<0.01, ***p<0.001).

**
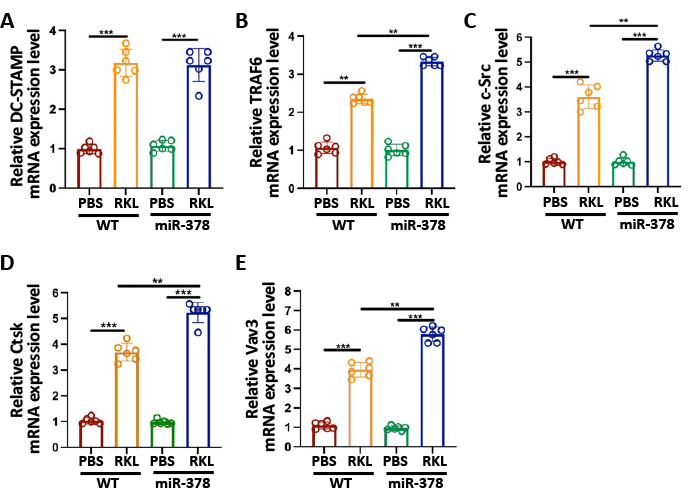
**

**Supplementary Figure 2.** The mRNA expression level of osteoclastogenic differentiation related markers in WT and miR-378 BMNCs after treated with RANKL for 5 days, including DC-STAMP (A), TRAF6 (B), c-Src (C), Ctsk (D) and Vav3 (E) measured by qRT-PCR (n=6; *p<0.05, **p<0.01, ***p<0.001).


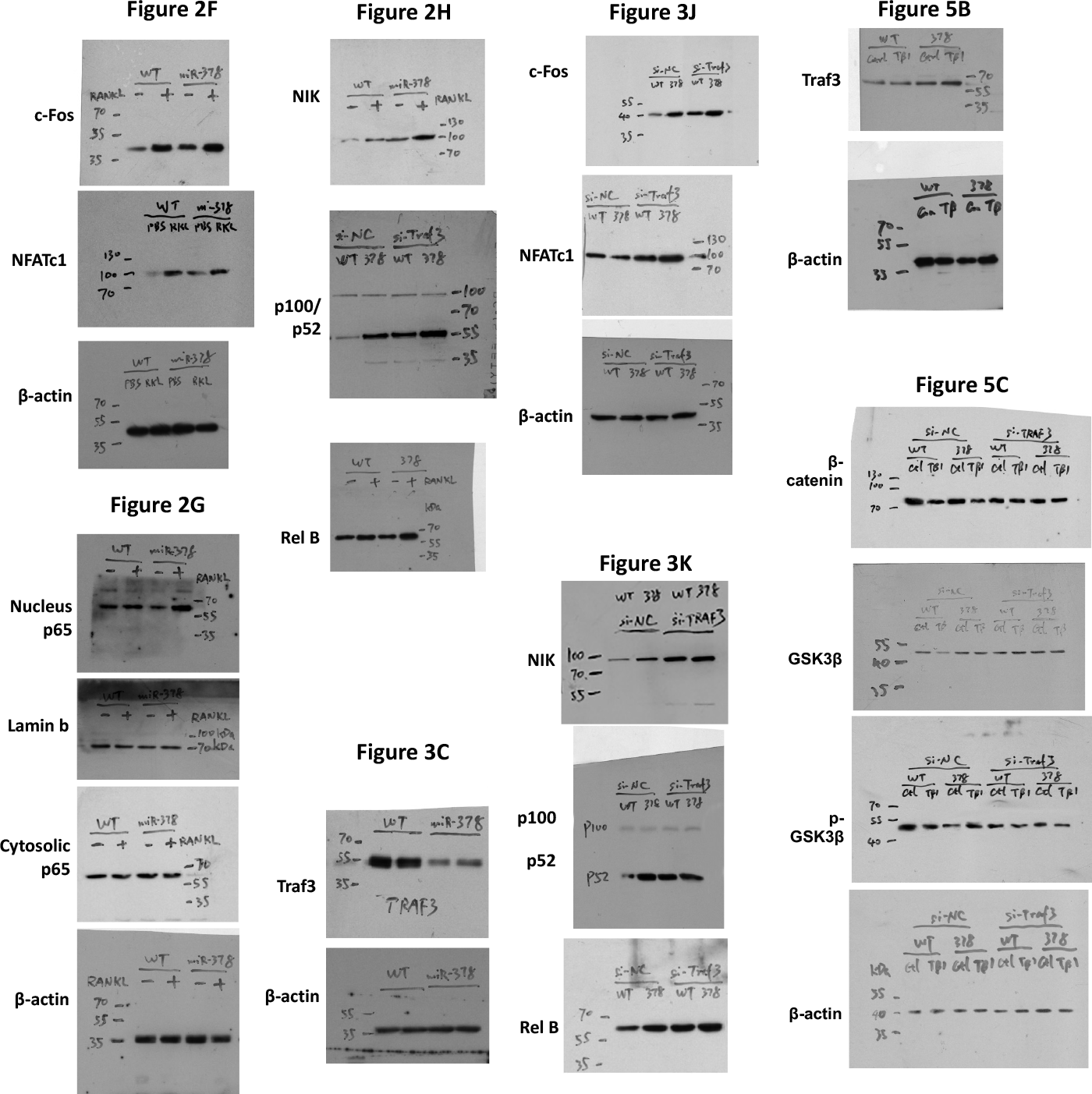


**Supplementary Figure 3**. **Full length uncropped original Western blots used in the manuscript**
